# Characterization of gluten‐free cupcakes without sucrose based on defatted soybean flour and monk fruit

**DOI:** 10.1002/fsn3.3840

**Published:** 2023-11-20

**Authors:** Mahshid Bahraminejad, Omid Rostami, Mahshid Heydari, Mohammadhadi Moradian, Khadije Abdolmaleki

**Affiliations:** ^1^ Student Research Committee, Department of Food Science and Technology, School of Nutrition Sciences and Food Technology Kermanshah University of Medical Sciences Kermanshah Iran; ^2^ Department of Food Science and Technology, Faculty of Nutrition Science and Technology/National Nutrition and Food Technology Research Institute Shahid Beheshti University of Medical Sciences Tehran Iran; ^3^ Research Center of Oils and Fats Kermanshah University of Medical Sciences Kermanshah Iran

**Keywords:** *Cydonia oblonga*, defatted soybean flour, gluten‐free cake, monk fruit, *Plantago ovata*

## Abstract

This study aimed to produce and characterize a novel gluten‐free cupcake for celiac and diabetes people. For this purpose, wheat flour and sugar in the cupcake formulation were fully replaced with soy flour and monk fruit. Also, samples containing wheat flour with sugar and monk fruit were prepared for comparison. The gluten‐free cupcake without sucrose had a less specific volume and porosity index. To improve these properties, *Cydonia oblonga* (Cydonia Vulgaris) and *Plantago ovata* (Plantago genus) were used individually and in combination at concentrations of 1 and 2%. The cake containing no gum was made as a control as well. It was observed that addition of gums had positive effects on the specific volume, porosity index, and weight loss of cakes, but their incorporation increased their hardness. Based on the results, the fabrication of a novel and successful gluten‐free cupcake replaced with soy flour, monk fruit, and gum is possible.

## Graphical abstract

The development of gluten‐free bakery products has a growing market worldwide. Food scientists are trying to improve the quality of these products. The results of the present study revealed the capability of defatted soybean flour and monk fruit for utilization in formulations of sugar‐ and gluten‐free cakes, improving their nutritional value and quality characteristics. The results of this study could be useful and applicable for the bakery industry.

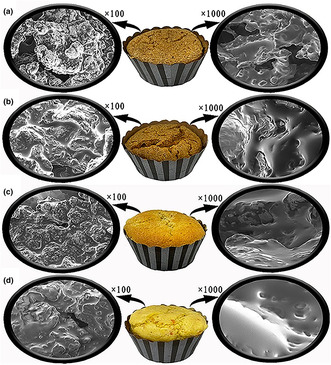

## 1 INTRODUCTION

Cakes are classically sweet baked desserts with flexible and elastic crumbs commonly made from wheat flour. It is a widespread and staple food due to its availability in various types and reasonable cost, used by approximately all people in society (Sicherer & Sampson, 2014). Traditional cupcakes are made with low‐gluten flour containing less than 9% gluten protein and are delicious cakes with high nutritional value. Forming a cohesive protein network is essential for the flour batter's anticipated viscoelasticity. The agreeable network structure in the base dough is vital to trap the maximum released gas through the baking and reach the best expansion in texture. When water is added to the mixture of wheat flour, the viscosity and extensibility are because of the prolamin fraction, while the elastic and sticky properties of the dough are introduced by the glutenin fraction. There is a truth about these complimentary desserts and other products based on wheat flour; the cupcake may be dangerous for celiac disease. Celiac disease is a chronic small bowel malabsorption syndrome as a result of the patient's intolerance to gluten protein, and so the demand for gluten‐free products is increasing (Gao et al., 2018). In addition, with the spreading knowledge of dietary sugar consumption and related chronic diseases (type II diabetes and obesity), the demand to reformulate food products to decrease refined sugar content is imperative. The first candidate to investigate substitute sucrose replacers is confectionery products due to their significant sucrose functionality and desired organoleptic and structural properties. In the past, sucrose substitute/reduction mostly involved artificial sweeteners like sugar alcohols (polyols), owing to their capability to mimic sucrose on the subject of sweetness and functionality (Rippe & Angelopoulos, 2013).

In recent years, the food industry has tended to fortify traditional foods that people consume heavily, increasing their nutritional value and turning them into functional foods, such as fortified bread with some legumes, including soybeans, chickpeas, and lupine (Mohammed, 2019). Also, this food can be a vital source of stroke prevention, type II diabetes, vascular disease, and gastrointestinal cancer because of its high fiber, vitamins, and minerals. Despite the recent advances in the formulation of gluten‐free products with similar quality, the full gluten replacement in the products based on cereal, such as bread, cake, biscuits, pasta, and breakfast cereals, is a serious challenge. On the other hand, flours obtained from various plant sources are considered for partial or total replacement with wheat flour. However, the substitution of wheat flour typically leads to a reduction in the quality of the product (Di Cairano et al., 2022; Queiroz et al., 2022). Soybean meal (SBM) is a protein supplement used in dairy rations and is the standard for determining the value of other protein supplements. SBM is very palatable and contains large amounts of essential amino acids (approximately 45% of total amino acids) except for sulfur‐containing ones like methionine. Its protein digestibility score is about 1, which is close to some of the proteins from animal sources, such as dairy and meat, which can be substituted with wheat flour in the cake (Bernard, 2016; de Oliveira Silva et al., 2018; Twinomuhwezi et al., 2020).

Monk fruit consists of a group of triterpenoid glycosides, which are regarded as the main active components of the sweet taste and responsible for the main biological functions of monk fruit. Monk fruit has been shown to have health benefits including anti‐asthmatic, antitussive, glucose‐low‐erring, antioxidative, liver‐protective, immunoregulating, and possibly anticarcinogenic properties (Ban et al., 2020). Also, it is one of the most popular sweeteners in the United States that is safe, low‐calorie, and suitable for people with type 2 diabetes. Monk fruit can be considered a substitute for sugar (Harshita, 2023).

Adding additives to baked products usually involves changes in their constructive infrastructure properties, affecting final product quality. Hence, the researcher's score for delicious cakes primarily focuses on the batter or dough's rheological properties (Noorlaila et al., 2020).

Salehi (2019) investigated the use of natural hydrocolloids to improve the properties of gluten‐free bread and cakes. The results of this study showed that the addition of gum to baked products, based on their ability to interact with water, affects the crumb softness and increases their volume, porosity, and shelf life (Salehi, 2019). However, some studies have shown that the presence of gum in the formulation, along with other compounds such as emulsifiers, may decrease the volume and increase the hardness of the texture (Noorlaila et al., 2020). Considering that the characteristics of gluten‐free cupcakes without sucrose using defatted soybean flour, monk fruit, *Cydonia oblonga* gum (Cg), and *Plantago ovata gum* (Pg) have not been investigated so far, this study aimed to reveal the effect of different types and percentages of gum addition on cupcakes’ physicochemical, nutritional, and sensory features to manufacture gluten‐free cupcakes with favorable properties. The effect of replacing sugar (sucrose) has also been investigated.

## 2 MATERIALS AND METHODS

### 2.1 Materials and gum preparation

Commercial soybean flour was provided by Behpak, Soybean Flour Co. (Iran, Behshahr). The flour was characterized as having 8.17% moisture, 4.07% ash, 1.25% fat, and 61.24% protein content. Commercial wheat flour (14% moisture, 0.79 fat content, and 13.2% protein content) was provided by Khoshe Gandom, Co. (Iran, Kermanshah). Other ingredients such as rose water, sunflower oil, carrot puree, cinnamon, baking powder, egg, milk, salt, and monk fruit extract (25% mogroside) were purchased from the local market (Iran, Kermanshah). The Cydonia oblonga *(Cydonia Vulgaris*) and Plantago ovata (*Plantago genus*) were harvested at the end of spring from the mountains of Kermanshah, Iran. For gum preparation, 1000 g of Cydonia oblonga seed were put into 1 liter of water for 5 h to release the mucilage, and then a fabric filter was used to separate the solution phase. In the last step, alcohol (Ethanol) 95% was added to extract the gum. Afterward, the precipitated mucilage was dried in an oven (Memmert – Germany) at 50°C for 48 h. The Plantago ovata seeds were ground in milling (Best – Taiwan). The ground Pg seeds are used directly in the cake formulation.

### 2.2 Cupcakes production

Following the method described by Noorlaila et al. (2020) with minor modifications in the formula, cupcakes were prepared according to Table 1 with the addition of Cg and Pg at 1 and 2% concentrations. To mimic commercial confectionery, a mixture of soybean flour, egg, and hydrated hydrocolloid (based on the sample preparation) was mixed in a Hobart mixer (Dessini DS‐268, Italy). Then, a constant amount of oil, milk, carrot puree, baking powder, rosewater, sugar, or monk fruit as a sweetener was added to the mixtures. All ingredients were mixed for 1 min at high speed (no. 4) until a uniform and soft batter mass was obtained. Finally, samples were divided into a rounded ball shape in a mold and baked in the oven (Revent, US) for 40 min at 160°C. After cooling for 30 min, the cakes were packed in polypropylene bags at room temperature for further analysis. In this research, there were 10 samples, as follows: Control (including soy flour (SF) and monk fruit (MF) without Cg and Pg), Cg1 (including SF and MF with 1 wt.% Cg), Cg2 (including SF and MF with 2 wt.% Cg), Pg1 (including SF and MF with 1 wt.% Pg), Pg2 (including SF and MF with 2 wt.% Pg), CPg1 (including SF and MF with 1 wt.% Pg and 1 wt.% Cg), CPg2 (including SF and MF with 2 wt.% Pg and 2 wt.% Cg), WS (including wheat flour (WF) and sugar), WM (including WF and MF), and SS (including SF and sugar).

**TABLE 1:** The ingredients used in cupcake formulation.

Ingredients (gr)	Control	Cg1	Cg2	Pg1	Pg2	CPg1	CPg2	WS	WM	SS
SF	100	100	100	100	100	100	100	0	0	100
WF	0	0	0	0	0	0	0	100	100	0
MF	1.33	1.33	1.33	1.33	1.33	1.33	1.33	0	1.33	0
Sugar	0	0	0	0	0	0	0	85	0	85
Pg	0	0	0	1	2	0.5	1	0	0	0
Cg	0	1	2	0	0	0.5	1	0	0	0
Rose water	33.33	33.33	33.33	33.33	33.33	33.33	33.33	33.33	33.33	33.33
Sunflower oil	50	50	50	50	50	50	50	50	50	50
Carrot puree	33.33	33.33	33.33	33.33	33.33	33.33	33.33	33.33	33.33	33.33
Cinnamon	0.16	0.16	0.16	0.16	0.16	0.16	0.16	0.16	0.16	0.16
Baking powder	3.33	3.33	3.33	3.33	3.33	3.33	3.33	3.33	3.33	3.33
Milk	96.6	96.6	96.6	96.6	96.6	96.6	96.6	96.6	96.6	96.6
Salt	0.1	0.1	0.1	0.1	0.1	0.1	0.1	0.1	0.1	0.1
Egg	50	50	50	50	50	50	50	50	50	50
Total	368.18	369.18	370.18	369.18	370.18	369.18	370.18	451.85	368.18	451.85

### 2.3 The proximate composition

The ash was evaluated by burning 2–3 g of the samples in a muffle furnace as recommended by the (AOAC, 2000). The amount of protein was calculated by the Kjeldahl method as recommended by the (AOAC, 1995). The conversion factor of nitrogen to protein was 6.25. The crude fat was determined by extracting 2–3 g of the sample in hexane (boiling point, 69°C) in a Soxhlet extractor (AOAC, 2000). The AACC (2000) standard, 16–44 number was used to measure moisture.

### 2.4 Porosity index

To evaluate the porosity, the image analysis technique was used. Images were taken by an HP scanner (HP Scanjet 2400, China) with a resolution of 300. The obtained images were evaluated using the software (Image J). By activating the 8‐bit part, gray‐level images were created. To convert gray images into binary images, the binary part of the software was activated. These images were a collection of light and dark points, and the ratio of light to dark points was estimated as an indicator of the sample's porosity. Finally, by activating the analysis part of the software, this ratio was calculated, and the porosity percentage of the samples was measured. It is obvious that the higher this ratio is, the higher the number of holes in the texture (porosity).

### 2.5 Specific volume

Cake volume was measured for 2 h after cooling. The volume of the cupcake was determined by the rapeseed displacement method (AACC Method 10–91), and the specific volume was obtained by the ratio of the cupcake volume to cake weight (AACC, 2000).

### 2.6 Weight loss

The weight loss (%) was determined by using the following equation. Cake weight loss was assessed on ten independent cake samples from each type for 24 h after baking.

$$\%\text{weight loss}=\frac{w_{0}-w_{f}}{w_{0}}\times 100$$

where *W*_*f*_ is the weight of the cake samples after baking and cooling, and *W*_0_ is the weight before baking (initial cake dough).

### 2.7 Texture analysis

The Texture Analyser (Testometric M350‐10CT, England) was used to determine the texture of cake samples (Texture Profile Analysis test (TPA)). This test records the force–time curve for two cycles of compression. All measurements were done at room temperature (25°C). The measurements were accomplished on the cake samples with dimensions of 60 × 60 × 40 mm. The samples were compressed to 50% (20 mm) of their original height with a displacement speed of 2 mm/s using an aluminum cylinder probe. Texture profile parameters, firmness (N), cohesiveness, adhesiveness, and springiness were assessed. Texture analysis was measured on three independent samples from each treatment 24 h after baking.

### 2.8 Cake microstructure

Optimized cupcake samples were mounted on sample holders using double‐sided sticky tape and detected using a scanning electron microscope (JEOLJSM‐5400, Japan) at a 15 kV accelerating voltage. Samples were sputter‐coated with 20 nm of gold using a JEOLJFC‐1100E ion sputter coating device. Micrographs were presented at a magnification of 100× and 1000×.

### 2.9 Sensory evaluation

The gluten‐free cupcakes prepared with different types and concentrations of gum and sweetener were evaluated by 20 semi‐trained panelists. The cake samples were cut into slices of the appropriate size (20 × 20 × 20 mm), including the center points, and then labeled with a three‐digit code to serve the panels randomly. The panelists were requested to evaluate cake samples based on their acceptance of their color, odor, texture, taste, and overall acceptability using a 5‐point hedonic scale that ranged from 1 (dislike extremely) to 5 (like extremely) for each organoleptic characteristic.

### 2.10 Statistical analysis

All experimental values are presented as mean ± standard deviation in triplicates for each test except for texture analysis (5 runs) and sensory evaluation. One‐way analysis of variance (ANOVA) and the Kruskal–Wallis test were applied to examine the differences between samples. Duncan's multiple range test (*p* < .05 probability level) was used to determine the significance of treatments. Statistical analysis of the data was performed using the SPSS (version 21) software.

## 3 RESULTS AND DISCUSSION

### 3.1 Moisture content

According to the investigations carried out and the results obtained from the statistical analysis of Table 2, the highest moisture content was related to the sample with the formulation of wheat flour and sugar (WS), and the sample with the formulation of 1% Pg had the lowest moisture content. In other samples in which the amount of soybean flour and sweetener was constant and only the type of gum and their percentages were different, it showed that the type of gum and their percentages had a significant effect on this parameter (*p* < .05). Also, the samples containing 2% Cg had a higher moisture content than other samples, which could be due to the high ability of gum to retain moisture. Salehi (2017) reported that the moisture content of apple cake formulated with wild sage seed gum increased with the addition of gum due to the increased capacity of hydrocolloids to absorb water and moisture retention (Salehi, 2017).

**TABLE 2:** The proximate composition and physical properties of cupcakes.

Sample	Ash (%)	Moisture content (%)	Weight loss (%)	Specific volume (ml g^−1^)	Porosity index (%)
Control	2.54 ± 0.03^e^	35.3 ± 0.01^h^	14.0 ± 0.01^c^	0.18 ± 0.01^e^	22 ± 0.05^f^
Pg1	2.65 ± 0.06^d^	34.8 ± 0.03^k^	12.5 ± 0.01^e^	0.19 ± 0.01^e^	22 ± 0.20^f^
Pg2	3.09 ± 0.03^b^	36.1 ± 0.03^d^	11.9 ± 0.01^j^	0.22 ± 0.02^d^	26 ± 0.02^d^
Cg1	2.54 ± 0.05^ed^	35.6 ± 0.01^f^	12.5 ± 0.03^e^	0.17 ± 0.02^e^	20 ± 0.05^j^
Cg2	3.02 ± 0.01^c^	36.2 ± 0.02^c^	11.5 ± 0.01^h^	0.36 ± 0.03^b^	29 ± 0.09^b^
CPg1	2.68 ± 0.25^ed^	35.5 ± 0.02^j^	12.1 ± 0.02^f^	0.37 ± 0.02^b^	28 ± 0.08^c^
CPg2	3.68 ± 0.30^a^	36.1 ± 0.01^d^	12.8 ± 0.01^d^	0.30 ± 0.02^c^	26 ± 0.07^d^
SS	2.54 ± 0.03^e^	35.8 ± 0.04^e^	13.9 ± 0.02^c^	0.11 ± 0.02^f^	22 ± 0. 30^f^
WS	2.14 ± 0.03^f^	38.0 ± 0.01^a^	14.40 ± 0.01^a^	0.49 ± 0.03^a^	32 ± 1.00^a^
WM	2.12 ± 0.03^f^	37.2 ± 0.01^b^	14.1 ± 0.01^b^	0.24 ± 0.03^d^	25 ± 0.01^e^

*Note*: Data are mean ± standard deviation in triplicate. Values with a similar lowercase letter are not significantly different (*p* < .05).

Abbreviations: Cg, *Cydonia oblonga gum*; Pg, Plantago ovata gum; SS, Sugar, and soy; WS, Wheat flour and sugar; WM, Wheat flour, and Monk.

### 3.2 Porosity index

**FIGURE 1:**
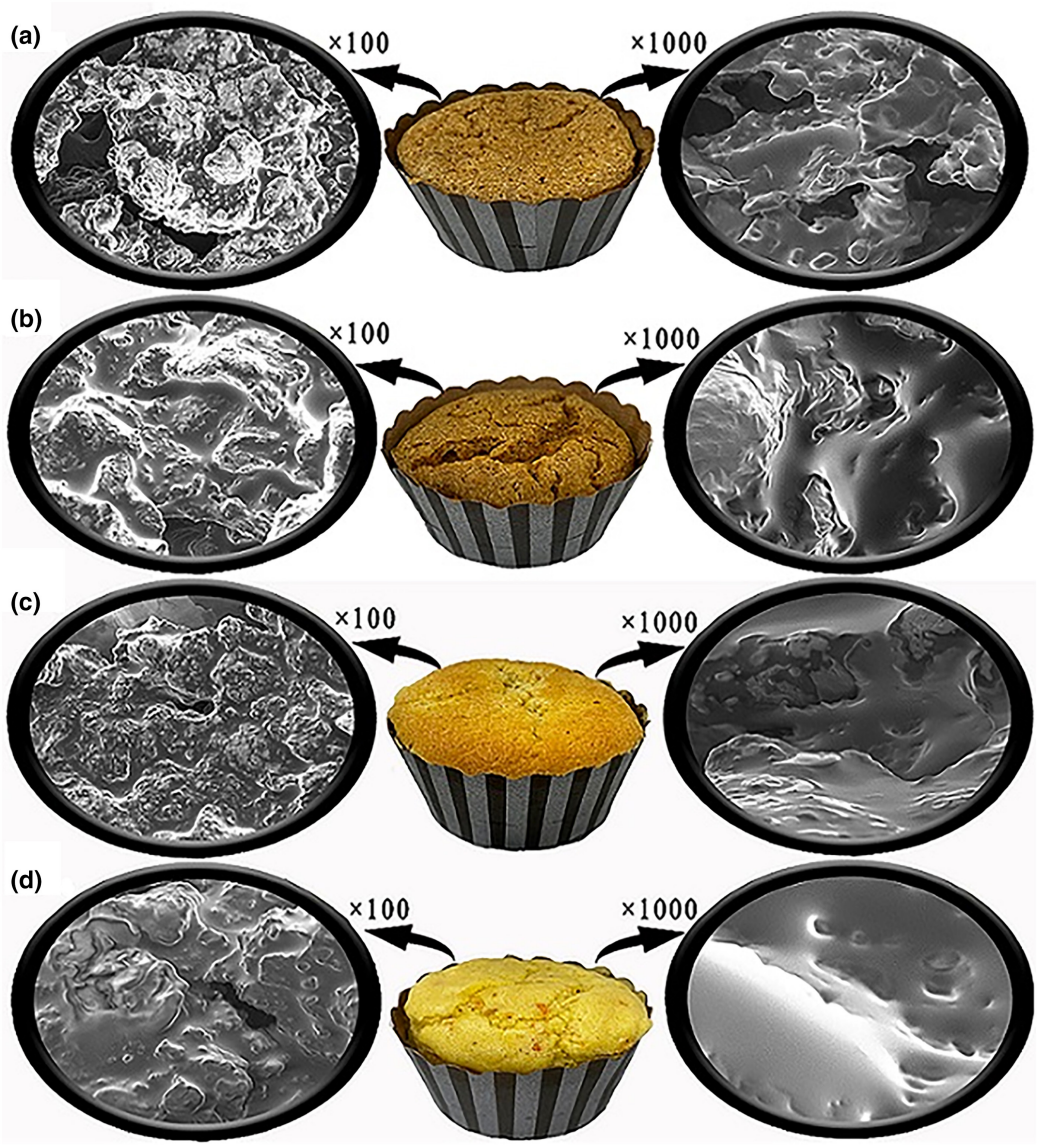
SEM micrograph (100× and 1000× magnifications) for cupcakes. a: SS, b: control, c: WS, and d: WM.

Porosity is a feature that demonstrates the ability to support the structure of the batter with trapped bubbles of carbon dioxide and air that are retained during the kneading process. The porosity index of the samples was in the range of 20.05%–32.10%. As shown in Table 2, the cupcake made with wheat flour and sugar (WS) has more porosity than the WM, control, and SS. This can be seen in Figure 1, in which C shows more porosity compared to the control (soy flour cake containing no gum). The use of monk fruit sweetener reduced the porosity index in the cupcake sample prepared with wheat flour, while the sweetener type (monk/sugar) did not affect the porosity of the soy flour cake. Moreover, Table 2 indicated that the addition of gum (Cg/Pg) significantly increased the porosity index of soy flour cake samples. The best porosity was related to samples Cg2, and the lowest porosity was related to the Cg1. Our findings are in agreement with those reported by Turabi et al. (2008), who also observed that rice cake samples containing gum have high porosity and show a good texture (Turabi et al., 2008).

### 3.3 Specific volume

Weight loss percentage, specific volume variation, moisture content, and ash between different cupcake formulations are shown in Table 2. According to these data and the images obtained by SEM, the volume of the SF sample is less than the WF cupcake sample because the SF cupcake has an irregular and purely protein structure; as a result, large holes are seen on the surface, and it also has a more compact surface. In the WF cupcake sample, the holes are covered with starch gelatinization and have uniform structures. The highest specific volume was related to the cake samples containing sugar and wheat flour. These results were similar to those of Chinma et al. (2010)). Also, the reason for this increase in specific volume can be attributed to the dilution of the cupcake batter in terms of gluten content, which is due to the lack of gluten in SF. This will cause air bubbles to disappear from the cake batter and reduce the volume (Gomez et al., 2010; Gómez et al., 2007). In the WF sample containing sugar and monk fruit, the sample containing sugar has a higher volume owing to the delaying of the starch gelation and increased water absorption in the batter. Monk fruit greatly reduces the viscosity of the batter, which is useful for the SF cupcake sample because the SF batter is very dense and has a high viscosity; however, this is not suitable for the WF sample. Lee et al. (2020) observed that the increase in d‐allulose content in pound cakes led to lower heights and volumes (Lee et al., 2020). In the presence of gum, Cg2, and CPg1 had the highest specific volume, with the highest value in the samples containing 100% SF. Salehi and Amin Ekhlas (2018) showed that gum addition (wild sage seed gum) increased the volume and porosity of the sponge cakes (Salehi & Amin Ekhlas, 2018). They also reported that this effect depended on the concentration and kind of hydrocolloid used.

### 3.4 Weight loss

The percentage of weight loss is the variation in weight before and after baking. Weight loss is an important parameter for structural changes in the cake. Gas escaping during the baking step can explain this loss. The weight loss of the gluten‐free cupcakes is shown in Table 2. Cupcake samples containing sugar showed a greater weight loss. Also, the WF cupcake showed a significant increase in weight loss compared to the control cupcake (*p* < .05). Soybean contains high amounts of fiber which increase water absorption and prevent the excretion of more moisture from the cupcake during baking. Therefore, the weight loss of SF cake was less. It is noteworthy that the cooking loss is reduced with the presence of Cg and Pg, and this effect was especially pronounced at Cg2. It is probably related to the microstructure of soybeans (Figure 1). Yi‐yuan Shao et al. (2015) reported that an increase in the binding capacity of proteins and hydrocolloids with water may cause a change in weight loss (Shao et al., 2015). Since SF cupcakes contain high amounts of protein, the binding of protein, gum, and water reduces the amount of weight loss compared to WF cupcakes.

### 3.5 Texture properties

A texture profile analysis of free‐gluten cupcakes was carried out to determine the main parameters of cake quality. Cake formulations presented significant differences in case firmness and cohesiveness parameters; however, an insignificant effect on springiness and adhesiveness was observed. The texture firmness is defined as the maximum force during the first compaction cycle, and cohesiveness is determined by the ratio of the positive force area under the second compression to that during the first compression (Sahagún et al., 2018). According to Table 3, firmness in the control sample significantly increased from 0.06 N to 0.14 N when wheat flour replaced soybean flour. These results may be associated with microstructure and flow behavior. The soy flour had a compact structure with numerous protein fragments on its rough surface. Also, similar results have been reported, which pointed out the lack of a coherent texture to store vapors and gases in soy cake texture (Sahagún et al., 2018). The texture results of the mogroside‐rich and sugar samples showed a cereal‐dependent effect (soy or wheat flour). Monk powder, compared with sucrose, significantly increased the gluten‐based cake's softness. However, the opposite results were observed in the case of gluten‐free cake. Reducing the viscosity of the batter in the case of soy batter has had a favorable effect on the final texture, but the result was the opposite in the case of wheat flour. It seems mogroside‐rich powder cannot similarly influence soy starch gelatinization. According to microstructure, the matrix of the gelatinized starch and the coagulated proteins with a more expressed layered structure were observed in the wheat‐sugar sample. In contrast, the wheat–monk powder of the partly gelatinized starch and coagulated proteins was covered parallel to the air‐pocket walls and kept their fine structure to a greater extent. Alongside firmness data, cohesiveness was another TPA parameter that measured the strength of the internal bonds in the samples. This parameter is also a measure of how much the sample breaks in the consumer's mouth and hand (Mihaylova et al., 2021). In the monk–soy sample, mogrosides improved internal soy substructure interaction; however, many structure gaps in the surface made it impossible to preserve the produced gases. Table 3 results showed that the Cg in both concentrations (1 and 2% w/w) has a positive effect on gluten‐free cake firmness, significantly reducing the hardness. However, these results were not observed in the Pg case.

**TABLE 3:** Texture parameters of cupcakes manufactured with and without hydrocolloids and sugar.

Sample	Firmness	Cohesiveness	Springiness	Adhesiveness
	(*N*)	(−)	(−)	(N.s)
Control	0.14 ± 0.03^ab^	0.58 ± 0.08^b^	1.06 ± 0.10^a^	0.03 ± 0.03^a^
Pg1	0.09 ± 0.00^b^	0.66 ± 0.02^b^	0.99 ± 0.00^a^	0.02 ± 0.01^a^
Pg2	0.10 ± 0.03^b^	0.60 ± 0.06^b^	0.85 ± 0.26^a^	0.03 ± 0.03^a^
Cg1	0.16 ± 0.02^a^	0.81 ± 0.02^a^	0.99 ± 0.00^a^	0.03 ± 0.00^a^
Cg2	0.18 ± 0.02^a^	0.80 ± 0.01^a^	0.68 ± 0.37^a^	0.06 ± 0.06^a^
CPg1	0.11 ± 0.01^b^	0.64 ± 0.01^b^	0.92 ± 0.13^a^	0.02 ± 0.02^a^
CPg2	0.17 ± 0.02^a^	0.79 ± 0.01^a^	1.00 ± 0.01^a^	0.02 ± 0.01^a^
SS	0.05 ± 0.00^d^	0.49 ± 0.00^c^	0.98 ± 0.04^a^	0.02 ± 0.02^a^
WS	0.10 ± 0.03^b^	0.65 ± 0.00^b^	1.00 ± 0.04^a^	0.09 ± 0.06^a^
WM	0.06 ± 0.00^c^	0.48 ± 0.01^c^	0.99 ± 0.00^a^	0.02 ± 0.02^a^

*Note*: Data are mean ± standard deviation in 5 runs. Values with a similar lowercase letter are not significantly different (*p* < .05).

### 3.6 Macro and microstructure

**FIGURE 2:**
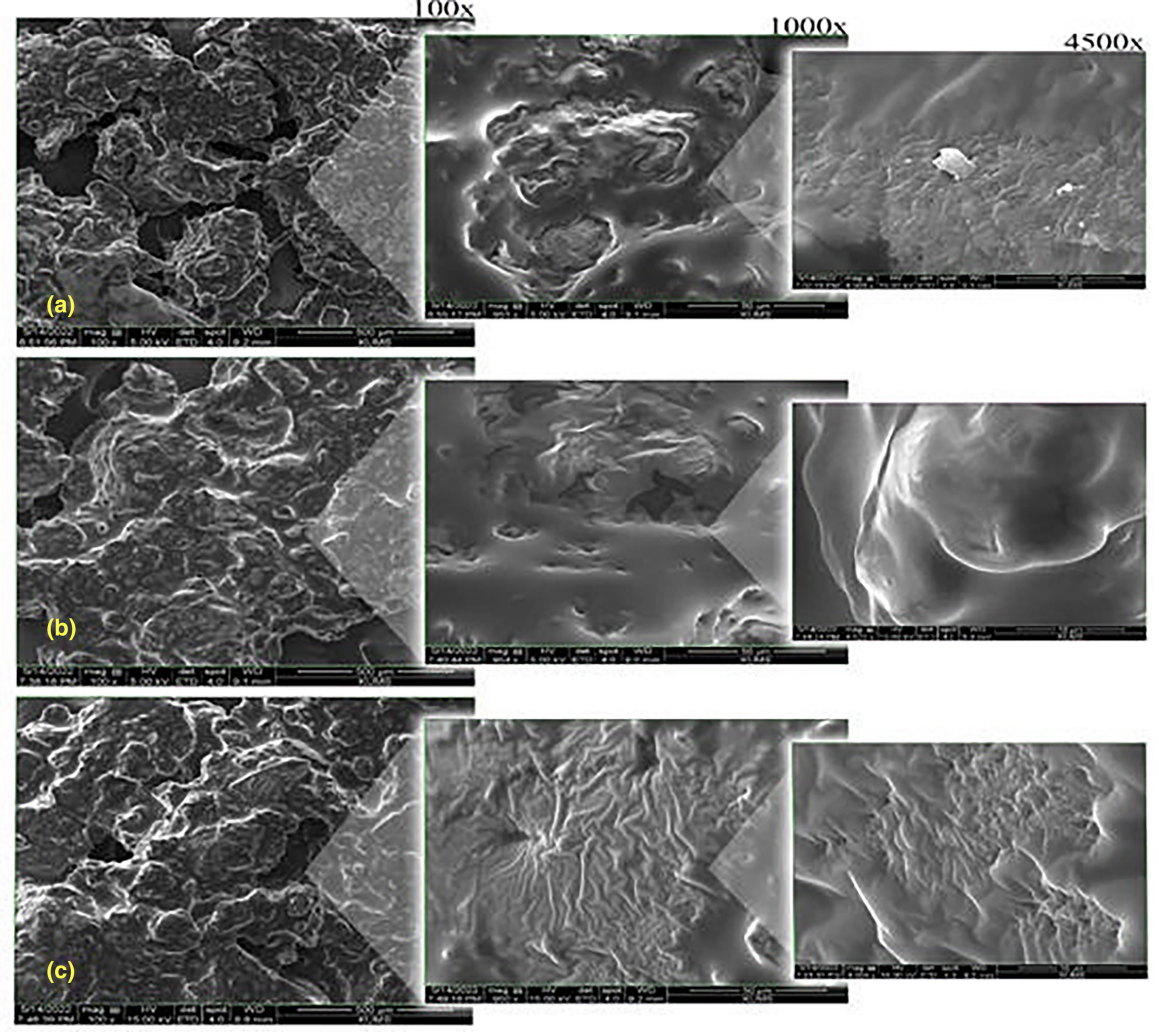
The SEM micrograph of gluten‐free samples. a: Pg2, b: Cg2, and c: CPg1.

The microstructures of gluten‐free soybean cake crumbs containing different sweeteners and gums were analyzed. A scanning electron microscope (SEM) has been obtained to study this surface property at 100× and the form of the bubble surface at 1000× and 4500× magnification (Figures 1 and 2). The sample information was based on the pore size distribution, the presence of irregular structures, and the covering particle properties. According to Figure 1 macrostructure observations, adding soybean flour to the cupcake recipe led to a compact texture with large holes at the bubbles’ surface. In the slice of cake, the diameter of the pores increased and, at the same time, decreased the density of the pores. Microstructure observation of soybean flour displayed a rough surface with many tiny pores and protein particles. The surface particulates in soybean flour samples were found to be correlated with protein fractions and have been previously reported (Dhen et al., 2016). On the opposite side, the wheat flour (Figure 1c) showed a flattened and smoother surface with coalesced pores in the homogenous distribution of cupcake bubbles (which was evident at more than 100x magnification). The cake batter based on wheat flour had a comprehensive and cohesive texture due to a complex network of ingredients including egg white, oil, gluten, starch, water, and more. This sample with a high level of starch had strong gelatinization, which affected the final formation of the porous structure. It seemed that tiny pores were masked and not visible because a continuous swollen matrix of starch covered them (Dhen et al., 2016; Goranova et al., 2019).

In addition to the debate on the base type of cakes, whether wheat or soybean, the effect of monk powder (mogrosides‐rich powder) by creating a cover sheet on the surface has a similar impact on both cake types (Figure 1b,d). According to Baeva et al. (2003), sucrose was essential in starch gelatinization and protein network development, restricting water during baking (Baeva et al., 2003). At the end of the baking period, sucrose played a role as a retarder of starch gelatinization. The air bubbles have enough time to expand from the carbon dioxide and water vapor before the batter solidifies. According to the specific volume section, the mogroside‐rich powder decreased the specific volume compared to the wheat–sugar sample but increased the volume in free‐gluten cupcakes. It seems that the monk powder could not delay the desired gelatinization of the cake. Also, both batter systems (wheat or soy) observed a significant decrease in batter viscosity. However, this decrease in thickness in soy batter was desirable due to its compact structure and undesirable for wheat batter due to increasing the escape of gas from the dough surface. The impact of Pg and Cg gums on bubble structure is shown in Figure 2. At 100× magnifications focusing on particle connection, the high concentration of Pg caused structure compression compared to the control sample. At the same time, the surface of the particles was slightly covered, but an irregular shape was still observed. At 4500× magnification, Pg appears to be well integrated into the protein structure. However, it seems that the soybean pieces are not completely closed together. A more uniform structure was observed at high Cg concentrations, especially when magnification levels of 1000× and 4500× were considered. This structure can be confirmed by the smooth appearance of the surface. The presence of hydrocolloids affects gelatinization intensity by changing the water available in the system. Shifted water‐holding capacity can change the thermal transitions of starch, butter viscosity, voluminous, and bubble structure (Baeva et al., 2003; Goranova et al., 2019; Hao et al., 2016). In previous research, similar results were reported for xanthan, guar, locust bean, and k‐carrageenan in the gluten‐free‐based due to the impact of gums on water absorption and rheological parameters (Turabi et al., 2010).

### 3.7 Viscosity property

**FIGURE 3:**
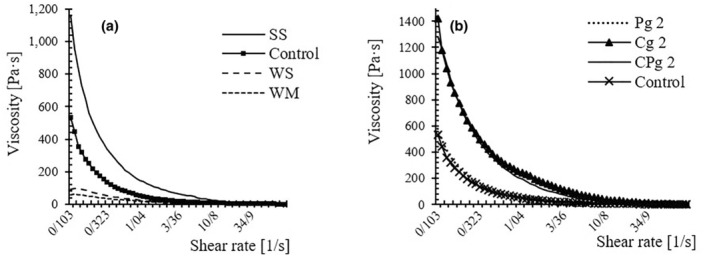
The viscosity property. a. Comparison between samples prepared with soy or wheat flour in the presence of sucrose and monk. b. Comparison between samples prepared with 2% w/w Cg, Pg, and a mixture of them.

The viscosity versus shear rate curve for different cake batters is shown in Figure 3. The batter matrix, known as a non‐Newtonian material at normal conditions, presents thixotropic properties and fluidity when mixed (3, 4). Non‐Newtonian material shows a stable structure at low stress, but the polymer structure at high strains is destroyed; as a result, viscosity decreases. All ingredients are mixed in one step in the all‐in‐one cake mixing method. The gluten network cannot be formed in this situation because of the high sugar and oil content and minor mechanical work during mixing (Lambrecht et al., 2018). Creatively, a series of experiments was designed to determine the impact of wheat replacement by soy flour on batter characteristics (Figure 3a). Soy samples at the initial shear rate have a higher viscosity than wheat samples. The increase in dough consistency and viscosity in the case of soy batter can be related to water‐holding capacity and the presence of oil in the raw material. The hydrophobic colloids act as anti‐foaming agents and promote bubble coalescence in batter, in which more giant bubbles quickly disappear on the surface of the slurry batter (Jia et al., 2014). Sucrose does not directly contribute to lowering surface tension (such as the role we envision for proteins, emulsifiers, or colloids as tension reducers), but indirectly playing as a stabilizer can reduce surface tension and overall diminish viscosity (Van der Sman & Renzetti, 2021; Wang et al., 2022). The results were much more intense for mogrosides‐source and a strong reduction in viscosity was observed in both samples with or without gluten. Investigating the behavior of gums, which were added to improve the rheological properties of free gluten batter, showed a strong and significant effect for Cg; however, the effect of Pg was insignificant.

### 3.8 Sensory evaluation

**FIGURE 4:**
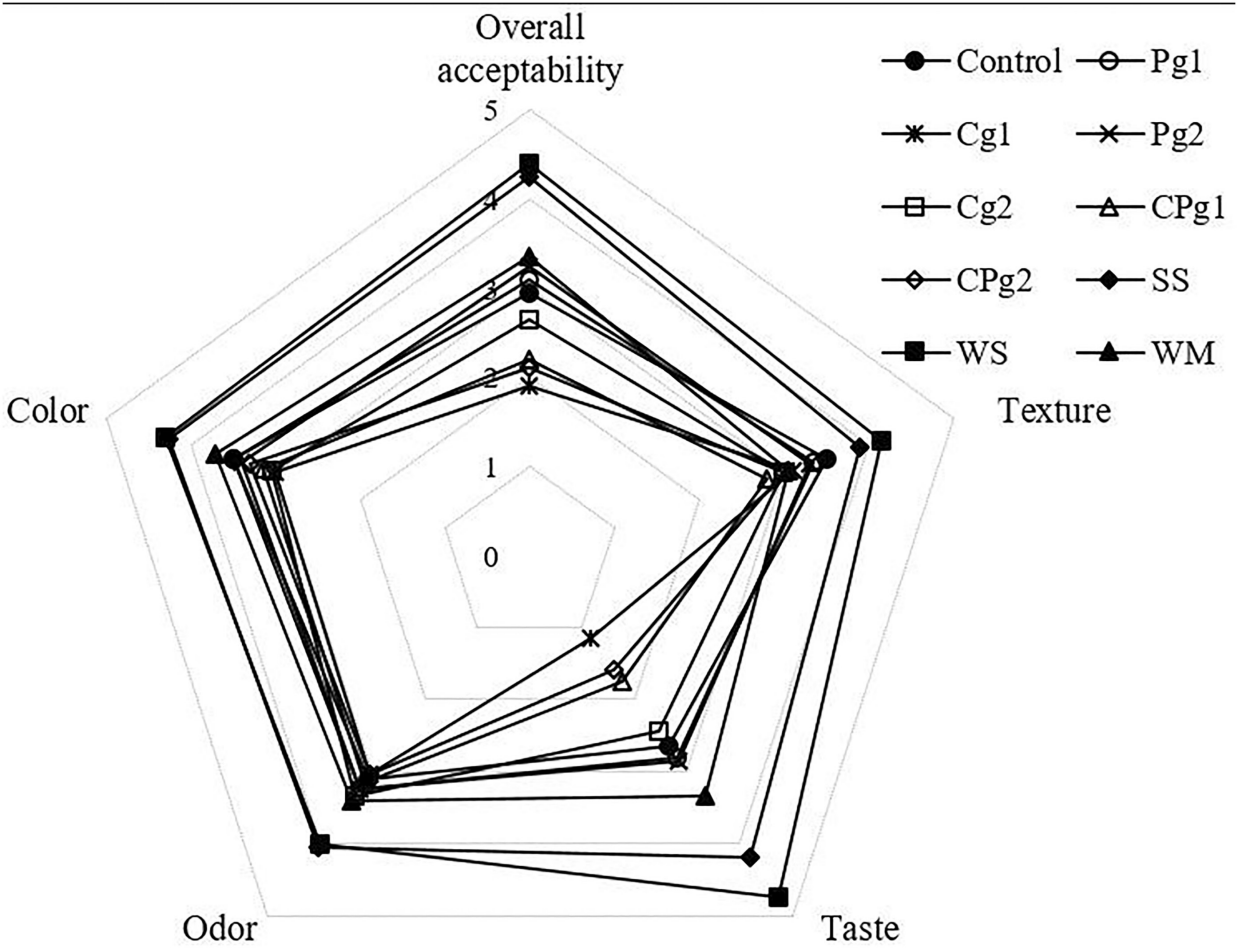
Radar map showing scores of color, odor, taste, texture, and overall acceptability from the sensory properties of cupcakes.

Appearance features such as taste and texture are the most important attributes for the development of a novel product. The scores of the five parameters (color, odor, taste, texture, and overall acceptability) were listed in the radar map (Figure 4). In all characteristics evaluated, wheat flour and sugar (WS) had the highest score. Those results were expected, as wheat flour and sugar are widely used in the bakery industry and consumers are familiar with their sensory attributes. SS was second in preference. This result may be related to the overall acceptance of sugar sweetness over monk fruit sweetness. After these samples, the WM sample also had high scores except for the texture, followed by Pg1 and Pg2, which had the highest overall acceptability compared to other samples. In general, there was no significant difference (*p* < .05) in terms of color and odor between treatments containing gum, but they were different in terms of texture and taste and, as a result, overall acceptance. In terms of taste, Pg1 and Pg2 received the highest scores, and the samples Cg1, CPg1, and CPg2 received the lowest scores. As previously discussed, the addition of different gums has a significant impact on the baking properties of the cake. In a study conducted by Salehi (2017), wild sage seed gum addition increased the volume and porosity of the cakes and resulted in softer products. Increasing gum levels significantly increased the crumb color and texture scores of apple cakes, according to a sensory evaluation (Salehi, 2017). However, in another study, the cake containing xanthan gum was the least acceptable because it increased the hardness of the cake, which contributed to its low overall acceptability by the panelists (Noorlaila et al., 2020).

## 4 CONCLUSIONS

This study aimed to develop a gluten‐free cupcake without sucrose with the same quality as a cupcake with sugar and wheat flour. This type of cupcake is made from ingredients such as soy flour and monk fruit sweetener as a substitute for wheat flour and sugar. In general, the specific volume of soy flour cupcakes was less than that of wheat flour. The reason was that the presence of holes in the microstructure caused moisture to escape, and as a result, the volume decreased. The use of monk fruit reduces the viscosity of the batter, which is desirable in soy cupcakes due to its high compactness and viscosity, but it is not desirable in wheat flour cupcakes. The addition of Cg and Pg had a positive effect on the cupcake at the level of 2% but increased hardness. There was a significant difference in the color, odor, and taste of the soy flour cupcake compared with the wheat flour cupcake. Among the gluten‐free samples, the formulation of using soy flour, monk fruit, and Pg as a substitute for wheat flour and sugar was most appreciated by the tasting panel. Therefore, a novel and successful formulation of gluten‐free cupcakes replaced with soy flour, monk fruit, and gum was developed.

## AUTHOR CONTRIBUTIONS

**Mahshid Bahraminejad:** Data curation (equal); writing – original draft (equal). **Mahshid Heydari:** Formal analysis (equal); investigation (equal); writing – original draft (equal). **Mohammadhadi Moradian:** Data curation (equal).

## CONFLICT OF INTEREST STATEMENT

All authors declare that they have no conflict of interest.

## ETHICS STATEMENT

This study does not contain any human or animal testing.

## Data Availability

The data that validate the results of this research are available from the corresponding author upon reasonable request.
